# The Human Neonatal Skin Fibroblast, an Available Cell Source for Tissue Production and Transplantation, Exhibits Low Risk of Immunogenicity In Vitro

**DOI:** 10.3390/ijms25136965

**Published:** 2024-06-26

**Authors:** Brice Magne, Karel Ferland, Étienne Savard, Martin A. Barbier, Amélie Morissette, Danielle Larouche, Chanel Beaudoin-Cloutier, Lucie Germain

**Affiliations:** 1 Department of Surgery, Faculty of Medicine, Université Laval, Québec City, QC G1V 0A6, Canada; 2 Centre de Recherche en Organogénèse Expérimentale de l’Université Laval/LOEX, Quebec City, QC G1J 5B3, Canada; 3 CHU de Québec-Université Laval Research Centre, Québec City, QC G1E 6W2, Canada; 4 Burn Care Unit, CHU de Québec-Université Laval Hospital, Québec City, QC G1J 1Z4, Canada

**Keywords:** fibroblast, immune response, transplantation, neonatal cells

## Abstract

The immunogenicity of allogeneic skin fibroblasts in transplantation has been controversial. Whether this controversy comes from a natural heterogeneity among fibroblast subsets or species-specific differences between human and mouse remains to be addressed. In this study, we sought to investigate whether fibroblasts derived from either adult or neonatal human skin tissues could induce different immune responses toward phagocytosis and T cell activation using in vitro co-culture models. Our results indicate that both phagocytosis and T cell proliferation are reduced in the presence of neonatal skin fibroblasts compared to adult skin fibroblasts. We also show that neonatal skin fibroblasts secrete paracrine factors that are responsible for reduced T cell proliferation. In addition, we show that neonatal skin fibroblasts express less class II human leukocyte antigen (HLA) molecules than adult skin fibroblasts after interferon gamma priming, which might also contribute to reduced T cell proliferation. In conclusion, this study supports the use of allogeneic neonatal skin fibroblasts as a readily available cell source for tissue production and transplantation to treat patients with severe injuries.

## 1. Introduction

Severe skin traumas, such as burns, are life-threatening for patients who are injured over extensive body surface areas. In order to shield these patients from infections and dehydration, rapid skin coverage must be achieved. Permanent coverage is completed using either skin autografts derived from patients themselves, or autologous skin substitutes produced in laboratories, such as ours [1]. Cell-free biomaterials can also be used to cover the wounds and help regenerate the dermis [2,3,4,5,6], but without a living epithelium, the skin barrier cannot be restored and patients can develop deadly infections. Allogeneic skin transplants derived from deceased donors can be a source of living cells; however, they are usually rejected a few weeks after transplantation [7,8,9]. The rejection of allogeneic skin grafts is caused by immunogenic cells residing within the grafts, including keratinocytes [10,11], endothelial cells [12], and immune cells [13,14], that express—or can be induced to express—high levels of human leukocyte antigens (HLAs) [15]. However, whether allogeneic fibroblasts participate in allograft rejection remains largely unknown. Allogeneic fibroblasts could yet be useful to accelerate the treatment of patients with critical injuries, as the isolation and amplification of autologous fibroblasts is a limiting factor for the production of several clinically approved tissue-engineered skin substitutes, including ours [16,17].

Fibroblasts consist of heterogeneous stromal cell subpopulations with tissue-specific characteristics [18,19], which can either promote or repress immune cell responses depending on their environment [20,21]. For example, skin fibroblasts can respond to toll-like receptor ligands or interferon (IFN) γ, through the release of chemoattractants, including CXC motif chemokine ligand 9 and 10 [22,23], that participate in the recruitment of Th1 lymphocytes, a T cell subtype known to precipitate allograft rejection [24,25]. In vitiligo, specific subpopulations of skin fibroblasts have also been shown to mediate auto-reactive CD8^+^ T cell recruitment in response to IFNγ [26]. However, fibroblasts can also exert immunosuppressive functions. In the gut, for example, fibroblasts inhibit the development of inflammatory macrophages through the release of prostaglandin E_2_ [27]. Lung macrophages can also induce monocytes to secrete more interleukin (IL)-10, an immune-suppressive cytokine, and less IL-12, a pro-inflammatory mediator [28].

In the context of transplantation, the role of allogeneic fibroblasts is also ambiguous. When used as a feeder layer for the production of cultured epithelial autografts (CEAs), allogeneic fibroblasts would induce a rapid second-set rejection in grafted mice [29], an outcome that our laboratory, however, never observed when treating patients with repeated series of CEAs (unpublished observations). Dermal substitutes produced from allogeneic fibroblasts have also been shown to be rejected by both innate and adaptive immune cells in grafted mice [30]. In humans, however, dermal substitutes produced from allogeneic fibroblasts combined with autologous skin grafts have been used to close permanently full-thickness burns without any signs of immune rejection [31]. In vitro, several studies using human cells have shown that allogeneic fibroblasts primed with IFNγ would not induce direct allorecognition by CD8^+^ T cells [32,33,34,35,36], but instead initiate indirect allorecognition by CD4^+^ T cells through prior contact with antigen presenting cells (APCs) [34,35,36]. Altogether, these contrasting pieces of evidence show that under specific circumstances, which are still barely understood, allogeneic fibroblasts may promote immune tolerance.

In order to obtain better insights into the immunogenicity of human allogeneic fibroblasts derived from diverse skin sources, we sought to investigate their interactions with different immune cells, including APCs, CD4^+^ and CD8^+^ T cells. Herein, we showed that neonatal skin fibroblasts—which can be isolated after parental informed consent from leftover tissues following surgery (e.g., foreskin circumcision or polydactyly corrective surgery)—express less class II HLA molecules than adult skin fibroblasts and secrete factors that reduce T cell proliferation. We also noted less phagocytosis of the neonatal skin fibroblasts compared to the adult skin fibroblasts. Overall, our findings suggest that allogeneic neonatal skin fibroblasts would be preferable to adult skin fibroblasts for potential transplantation applications.

## 2. Results

### 2.1. Phagocytic Activity of Antigen Presenting Cells Is Higher in Co-Cultures with Adult Skin Fibroblasts Than in Those with Neonatal Skin Fibroblasts

Since antigen presentation by APCs is the first step initiating CD4^+^ T cell-mediated immune responses [37], we first evaluated whether APCs could acquire fibroblast-derived membrane fragments after co-culture with human allogeneic fibroblasts. We thus isolated T cells and APCs from four different populations of PBMCs and confirmed their phenotype by flow cytometry (Figure S1A,B). Isolated T cell fractions contained nearly 50% of CD3^+^ HLADR^−^ T cells and less than 15% of CD3^−^ HLADR^+^ APCs, while isolated APC fractions contained nearly 75% of CD3^−^ HLADR^+^ APCs, with 55% of them being CD11c^+^ CD14low monocyte-derived dendritic cells (moDCs), a professional APC subset, and less than 6% of CD3^+^ HLADR^−^ T cells (Figure S1C).

**Figure 1:**
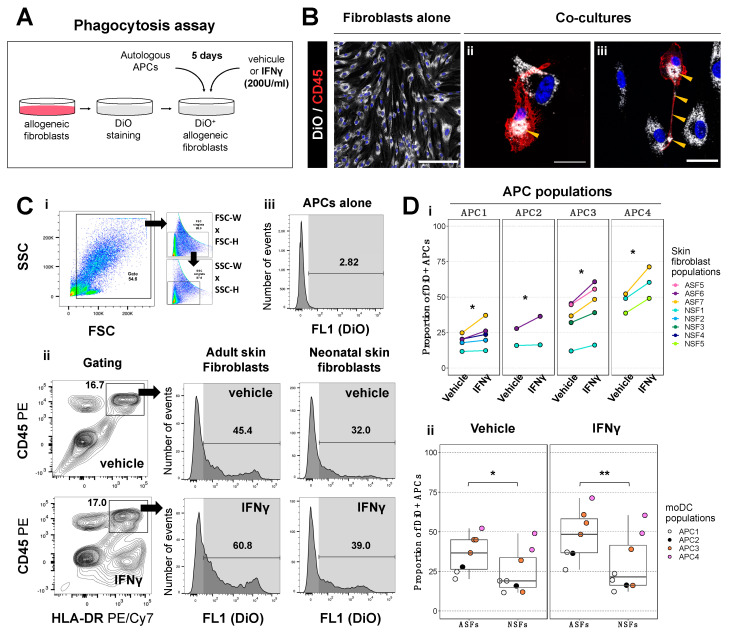
Phagocytosis is increased when human antigen presenting cells (APCs) are co-cultured with human adult skin fibroblasts or interferon (IFN) γ. (**A**) Schematic of the phagocytosis assay using the lipophilic fluorescent probe DiO. (**B**) Immunofluorescence of fibroblasts stained with DiO (shown in white), cultured (**i**) alone, or (**ii**,**iii**) with APCs stained with CD45 (shown in red). DAPI (shown in blue) depicts cell nuclei. Yellow arrowheads point to DiO incorporation by APCs. Representative images of APC2-ASF6 co-cultures. Scale bars: (**i**) 200 µm, (**ii**,**iii**) 15 µm. (**C**) Cytometric analysis of DiO incorporation by APCs. (**i**) Doublet exclusion strategy. (**ii**) Gating strategy to analyze DiO incorporation in CD45^+^ HLADR^+^ APCs co-cultured either with DiO^+^ Adult Skin Fibroblasts (ASFs) or DiO^+^ Neonatal Skin Fibroblasts (NSFs). Plots are generated on a biexponential scale. DiO expression is detected across the fluorescent detector channel 1 (FL1). (**iii**) Negative control of DiO incorporation by unlabeled APCs cultured without DiO^+^ fibroblasts. Representative graphs for APC3-ASF6 and APC3-NSF3 co-cultures are shown here. (**D**) DiO incorporation by moDCs expressed as the proportion of APCs that are DiO^+^ in APC-fibroblast co-cultures. (**i**) Data were segregated by APC donors and skin fibroblast populations across vehicle and IFNγ treatment conditions. (**ii**) The same data were represented after separating fibroblasts populations in two groups (ASF and NSF). Statistics: Wald test with Kenward-Roger’s multiple-comparison tests; * *p* < 0.05, ** *p* < 0.01, N = 15 different APC-fibroblast co-culture combinations.

We then co-cultured these four APC fractions with eight different allogeneic fibroblast populations, previously labeled with DiO, a lipophilic fluorescent probe (Figure 1A). Co-cultures were stimulated with IFNγ, a cytokine promoting both phagocytic activity and antigen presentation [38,39], or a vehicle (IFNγ dilution buffer). Using immunofluorescence, we visualized DiO^+^ fibroblast-derived membrane incorporation from dendrites to cytoplasm of CD45^+^ cells (Figure 1B, yellow arrowheads). However, we noted that DiO expression in stained fibroblasts only transiently localized to the plasma membrane, before being internalized to the perinuclear region (Figure S2, black arrowheads). Cytometric analysis further allowed us to quantify the proportion of CD45^+^ HLADR^+^ APCs that acquired DiO^+^ fibroblast-derived membranes (Figure 1C). As expected, IFNγ increased DiO^+^ fibroblast-derived membrane uptake by APCs, although the basal proportion of APCs that incorporated the fibroblast-derived membranes varied across APC populations (Figure 1D(i)). Interestingly, we noticed that IFNγ-mediated uptake of DiO^+^ fibroblast-derived membranes appeared higher with adult skin fibroblasts (ASFs) than neonatal skin fibroblasts (NSFs) (Figure 1C,f(i)). We therefore grouped all APC populations and compared DiO^+^ fibroblast-derived membrane uptake across ASF and NSF groups. Statistically, DiO^+^ fibroblast-derived membrane uptake was higher using ASFs than NSFs, irrespective of the vehicle or IFNγ condition (Figure 1D(ii)).

To determine whether membrane uptake reflected the phagocytic activity of APCs, we used DiO^+^ fibroblasts treated with camptothecin (CPT), a topoisomerase I inhibitor inducing apoptosis [40], or a vehicle (CPT dilution buffer), and co-cultured them with APCs (Figure S3A), as phagocytosis is triggered by apoptotic signals [41]. Compared to the vehicle, a 4.1-fold increase in membrane uptake was observed with CPT, even in the absence of IFNγ (Figure S3B,C). Moreover, CD45^+^ HLADR^−^ immune cells found in APC–fibroblast co-cultures (most likely T cells; see Figure S1) did not acquire DiO^+^ fibroblast-derived membrane fragments (Figure S3B,D), showing that this process was specific to the CD45^+^ HLADR^+^ cell fraction—i.e., APCs. We also investigated whether membrane uptake could result from extracellular vesicle transfer from DiO^+^ fibroblasts to APCs. To this end, we compared DiO incorporation by APCs cultured either with DiO^+^ fibroblasts (DiO-F) or their supernatant (DiO-SN) after IFNγ stimulation (Figure S4A). By immunofluorescence, membrane uptake in APCs was clearly visible in the DiO-F condition, while barely perceptible in the DiO-SN condition (Figure S4B). Using flow cytometry, we confirmed that DiO^+^ membrane uptake was not occurring when APCs were only incubated with the supernatant of DiO^+^ fibroblasts (Figure S4C,D). The data therefore suggest that the transfer of membranes was related to phagocytosis rather than cross-dressing—as observed in the semi-direct allorecognition pathway [37,42]. Overall, our data indicate that the phagocytic activity of APCs is increased in co-cultures containing ASFs compared to NSFs.

### 2.2. ASFs Induce More T Cell Proliferation Than NSFs

**Figure 2:**
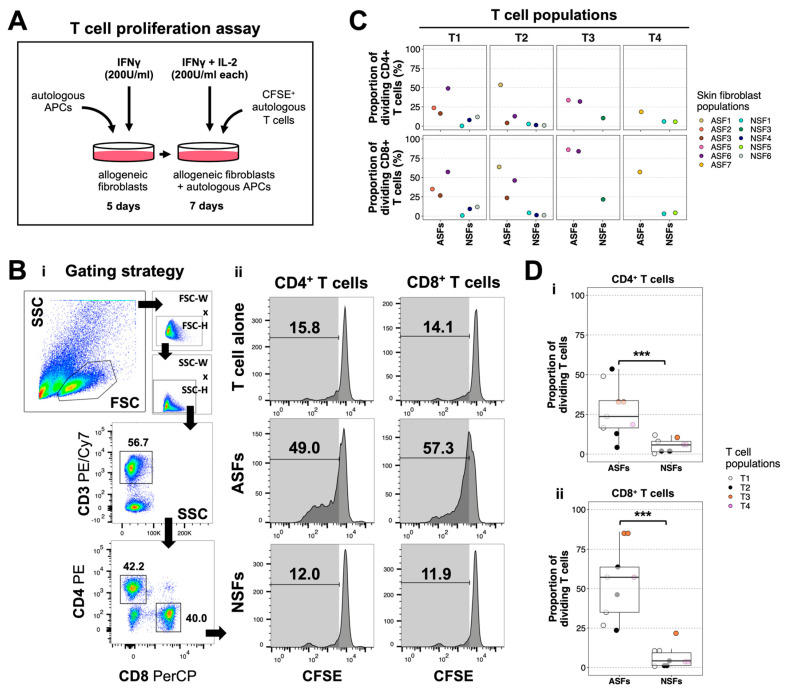
Alloresponsive T cell proliferation is increased in the presence of adult skin fibroblasts, but not neonatal skin fibroblasts. (**A**) Schematic of the T cell proliferation assay using carboxyfluorescein succinimidyl ester (CFSE) labeling. (**B**) Cytometric analysis of CD4^+^ and CD8^+^ T cell proliferation. (**i**) Gating strategy used to isolate CD4^+^ and CD8^+^ T cells. Plots are generated on a biexponential scale. (**ii**) Histograms showing CFSE expression in CD4^+^ and CD8^+^ T cell subsets across three different conditions: T cells alone, T cell-adult skin fibroblast (ASF) co-cultures, and T cell-neonatal skin fibroblast (NSF) co-cultures. Loss of CFSE expression indicate proliferation. The T cell alone control was used to set the gates defining the region of proliferating cells. Representative graphs for T1 alone, T1-ASF6 and T1-NSF6 co-cultures are shown here. (**C**) Proportion of dividing CD4^+^ and CD8^+^ T cells in co-cultures with N = 11 different skin fibroblast populations. Data were segregated by T cell donors (T1, T2, T3 and T4) and skin fibroblast populations (either adult-ASFs, or neontal-NSFs). (**D**) The same data were represented after separating fibroblast populations between ASF and NSF groups for CD4^+^ and CD8^+^ T cells. Statistics: ANOVA with Tukey’s multiple-comparison tests; *** *p* < 0.001, N = 18 different T cell-fibroblast co-culture combinations.

Because the phagocytic activity of APCs was weaker with NSFs than ASFs, we next wondered whether NSFs could also impact T cell proliferation. Accordingly, we stained T cells with CFSE, a proliferation tracer, and added them to autologous APC–allogeneic fibroblast co-cultures in the presence of IFNγ and IL-2 (Figure 2A). CFSE is a fluorescent probe which fluorescence intensity becomes weaker each time CFSE^+^ cells divide. Using flow cytometry, we evaluated CFSE fluorescence loss in CD4^+^ and CD8^+^ T cells isolated from co-cultures with APCs–ASFs or APCs–NSFs, or from cultures of T cells alone (Figure 2B). Using four different T cell populations, we screened 11 skin fibroblast populations, including six ASFs and five NSFs. The proportion of proliferating CD4^+^ and CD8^+^ T cells was usually higher in co-cultures with ASFs than with NSFs, but we noticed that some ASF–T cell combinations were less responsive than others (Figure 2C, see T2/ASF3). These differences might stem from variations in HLA mismatch between autologous T cells and allogeneic ASFs across all tested co-culture combinations. Moreover, we noticed that the proportion of proliferative cells was usually higher in the CD8^+^ than CD4^+^ T cell subsets (Figure 2C). To confirm these results, we grouped all T cell populations and compared the proportion of proliferating CD4^+^ and CD8^+^ T cells across ASF and NSF subgroups. Statistically, there were 3.8× more proliferating CD4^+^ and 13.2× more proliferating CD8^+^ T cells in co-cultures with ASFs compared to those with NSFs (Figure 2D). Notably, when we compared T cell proliferation in co-cultures of APCs, autologous T cells and either autologous or allogeneic fibroblasts, we noticed that only allogeneic ASFs induced significant proliferation of CD3^+^ T cells (Figure S5). Using a live–dead staining on fibroblasts co-cultured with T cells, we noticed—in a single experiment—that more dead adherent cells were found in co-cultures containing ASFs than NSFs (Figure S6), suggesting a weaker cytolytic activity of T cells in the presence of NSFs. Altogether, the data indicate that the proliferation of alloreactive T cells is higher in the presence of ASFs than NSFs.

### 2.3. NSFs Secrete Paracrine Factors That Reduce Alloreactive T Cell Proliferation

**Figure 3:**
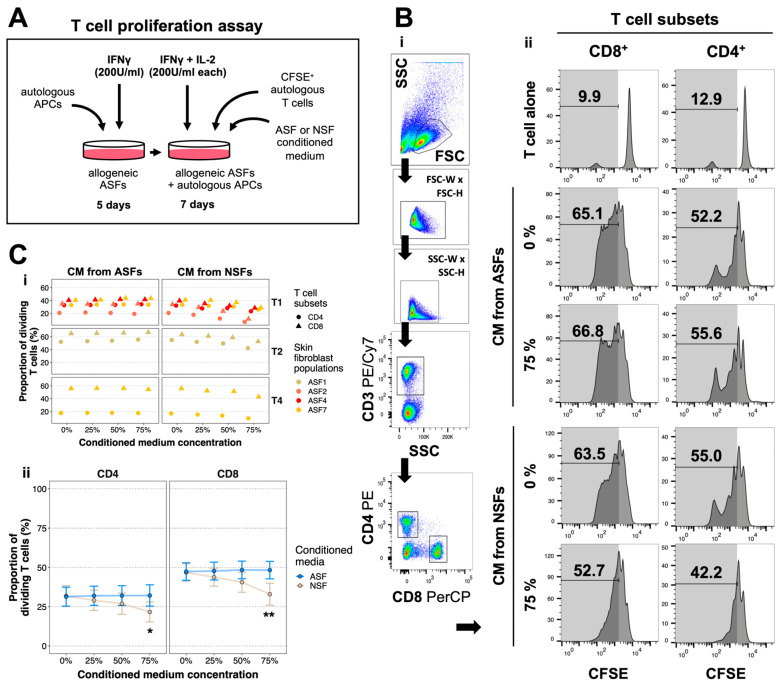
Conditioned medium from neonatal skin fibroblasts, but not from adult skin fibroblasts, attenuates alloresponsive T cell proliferation. (**A**) Schematic of the T cell proliferation assay using CFSE. (**B**) Cytometric analysis of CD4^+^ and CD8^+^ T cell proliferation. (**i**) Gating strategy used to isolate CD4^+^ and CD8^+^ T cells. Plots are generated on a biexponential scale. (**ii**) Histograms showing CFSE expression in CD4^+^ and CD8^+^ T cell subsets across three different conditions: T cells alone, T cell-adult skin fibroblast (ASF) co-cultures supplemented with conditioned medium (CM) from neonatal skin fibroblasts (NSFs), and T cell-ASF co-cultures supplemented with CM isolated from the same ASFs. Increasing doses of CM were added (0%, 25%, 50%, and 75%). Loss of CFSE expression indicates proliferation. The T cell alone control was used to set the gates defining the region of proliferating cells. Representative graphs for T2 alone and T2-ASF1 co-cultures are shown here. (**C**) Proportion of dividing T cells in N = 5 T cell-ASF co-cultures. (**i**) Data are segregated by T cell donors (T1, T2, T4), ASF populations (ASF1, ASF2, ASF4, ASF7), T cell subsets (CD4, CD8), and CM sources (from ASFs or NSFs). (**ii**) The same data were represented after separating CM sources between ASF and NSF groups for CD4^+^ and CD8^+^ T cells. Statistics: Wald test with Tukey’s multiple-comparison tests; * *p* < 0.05, ** *p* < 0.01, N = 5 different T cell-ASF co-culture combinations.

To uncover why ASFs appeared more immunogenic than NSFs, we next explored the role of their secretory products. We thus co-cultured allogeneic ASFs with autologous APCs and CFSE^+^ T cells and added conditioned media (CM) derived from either ASFs or NSFs at different concentrations three times a week for 7 days, along with IFNγ and IL-2 (Figure 3A). We then assessed CFSE fluorescence loss among CD4^+^ and CD8^+^ subsets by flow cytometry (Figure 3B), using five different ASF–T cell combinations. The proliferation of both T cell subsets was slightly reduced using increasing concentrations of CM derived from NSFs, but not from ASFs (Figure 3B(ii), C(i)). After segregating all data according to CM sources (ASF- or NSF-derived), we observed a dose-dependent reduction of T cell proliferation using CM derived from NSFs (Figure 3C(ii)). In particular, T cell proliferation in allogeneic ASF–T cell co-cultures was statistically reduced using high concentrations of NSF-derived CM (Figure 3C(ii), see 75%). However, T cell proliferation was not abrogated by the addition of NSF-derived CM as T cells alone were comparatively less proliferative (Figure 3B(ii)). Thus, ASF–T cell contacts or ASF secretion products may be responsible for maintaining T cell proliferation, even in the presence of NSF-derived CM, or NSF-derived CM alone may be insufficient to abrogate the proliferation of alloreactive T cells. Altogether, our data indicate that NSFs partially reduce alloreactive T cell proliferation through paracrine signaling.

### 2.4. Blockade of the NSF-Secreted Immune Suppressive Cytokine HLA-G Does Not Reduce T Cell Proliferation

**Figure 4:**
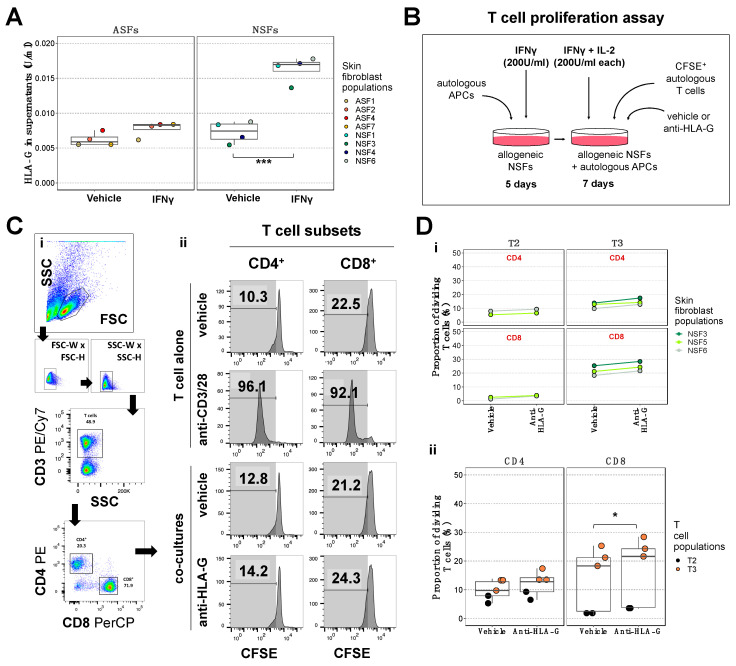
Blockade of neonatal fibroblast-derived HLA-G is necessary but not sufficient to suppress neonatal fibroblast-induced impairment of alloresponsive T cell proliferation. (**A**) Detection of HLA-G activity in concentrated supernatants of adult (ASFs) and neonatal (NSFs) skin fibroblasts after treatment with 200 U/mL interferon (IFN) γ or a vehicle. (**B**) Schematic of the T cell proliferation assay using CFSE. (**C**) Cytometric analysis of CD4^+^ and CD8^+^ T cell proliferation. (**i**) Gating strategy used to isolate CD4^+^ and CD8^+^ T cells. Plots are generated on a biexponential scale. (**ii**) Histograms showing CFSE expression in CD4^+^ and CD8^+^ T cell subsets across four different conditions: T cells alone with or without addition of anti-CD3/28 proliferation activators, and T cell-NSFs co-cultures supplemented with or without an anti-HLA-G neutralizing inhibitor. Loss of CFSE expression indicate proliferation. The T cells alone controls were used to set the gates defining the region of proliferating cells. Representative graphs for T3 alone and T3-NSF5 co-cultures are shown here. (**D**) Proportion of dividing T cells in N = 5 T cell-NSF co-cultures. (**i**) Data are segregated by T cell donors (T2, T3), NSF populations (NSF3, NSF5, NSF6), T cell subsets (CD4, CD8), and treatment conditions (vehicle or anti-HLA-G). (**ii**) The same data are represented after grouping all co-culture combinations across the two treatment conditions for CD4^+^ and CD8^+^ T cell subsets. Statistics: (**A**) Wald test with Tukey’s multiple-comparison tests; *** *p* < 0.001, (**D**) Wald test with Kenward-Roger’s multiple-comparison tests; * *p* < 0.05, N = 5 different T cell–NSF co-culture combinations.

In order to dissect the nature of the mediators involved in NSF immune-suppressive functions, we next assessed by ELISA the levels of HLA-G, a physiological immune checkpoint inhibitor [43], in NSF and ASF supernatants after a 12-day stimulation with IFNγ or a vehicle (IFNγ dilution buffer). While HLA-G levels were comparable in supernatant of both ASFs and NSFs treated with the vehicle, they were 2.3 times higher in NSF supernatants after IFNγ stimulation (Figure 4A). We therefore wondered whether the immune-suppressive activity of NSFs could be related to an increase in HLA-G production after IFNγ stimulation. To answer this question, we co-cultured allogeneic NSFs with autologous APCs and CFSE^+^ T cells in the presence of a neutralizing antibody raised against HLA-G (Figure 4B) or a vehicle (neutralizing antibody dilution buffer). We then assessed CFSE fluorescence loss among CD4^+^ and CD8^+^ subsets by flow cytometry (Figure 4C). Using five different NSF–T cell co-culture combinations, we noticed a very slight increase in T cell proliferation, especially in the CD8^+^ subset, using the neutralizing antibody (Figure 4D(i)). After grouping all co-culture combinations together, we confirmed that HLA-G inhibition in NSF–T cell co-cultures barely increased the proliferation of CD8^+^ T cells (Figure 4D(ii)). Because the addition of the neutralizing antibody did not trigger a strong T cell activation as observed in the control using anti-CD3/28 activators (Figure 4C(ii)), it is likely that NSFs either repress the immune response through other immune checkpoint inhibitors, or express less molecules that activate the immune system, such as those involved in antigen presentation.

### 2.5. NSFs Display a Phenotype That Could Facilitate Immune Evasion in Response to Inflammation

**Figure 5:**
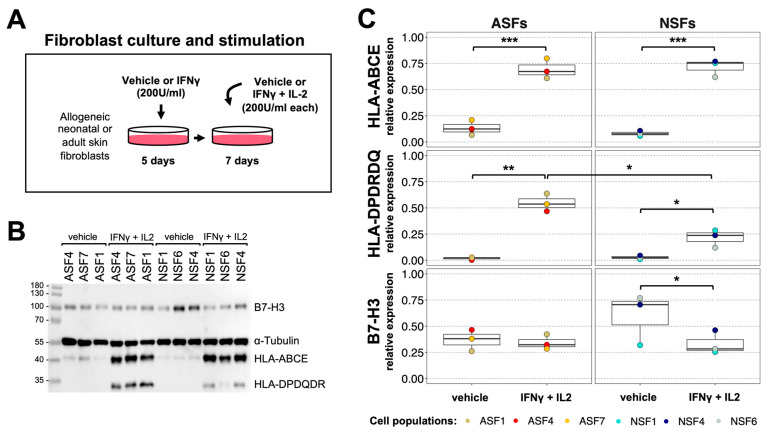
Neonatal skin fibroblasts primed with inflammatory signals express less molecules that activate the immune system. (**A**) Schematic of the protocol used to stimulate neonatal (NSFs) and adult (ASFs) skin fibroblasts. (**B**) Western blots on ASF and NSF lysates for the detection of HLA-ABCE, HLA-DPDQDR, B7-H3 and α-tubulin (used as a loading control). (**C**) Densitometry quantifications normalized to α-tubulin. Statistics: Wald test with Tukey’s multiple-comparison tests; * *p* < 0.05, ** *p* < 0.01, *** *p* < 0.001, N = 6 different fibroblast populations (3 NSFs and 3 ASFs).

Because HLA-G inhibition was not sufficient to abrogate T cell proliferation in NSF–T cell co-cultures (Figure 4), and since the addition of NSF supernatants to ASF–T cell co-cultures only partially decreased T cell proliferation (Figure 3), we next wondered whether there could be specific phenotypic differences distinguishing ASFs from NSFs. We therefore cultured three populations of ASFs and NSFs with IFNγ and IL-2 (Figure 5A) or a vehicle (IFNγ and IL-2 dilution buffer) and analyzed the expression of class I and II HLA molecules, as well as other co-stimulatory molecules in ASF and NSF lysates by Western blotting (Figure 5B). Interestingly, while the stimulation with IFNγ and IL-2 induced a strong expression of HLA-ABCE in both fibroblast sources, a significantly reduced increase in HLA-DPDQDR was observed in NSFs compared to ASFs (Figure 5C). Using flow cytometry, we also showed that HLA-DR surface expression was about 10 times lower in NSFs than ASFs after IFNγ treatment (Figure S7), indicating that surface antigen presentation is significantly reduced in NSFs after IFNγ induction. The expression of B7-H3, a co-stimulatory ligand for T cell activation and IFNγ production [44], also appeared higher in NSFs than in ASFs, but decreased significantly after IFNγ and IL-2 stimulation in NSFs only (Figure 5C). The data therefore indicate that NSFs regulate important surface protein expression in response to inflammatory primings.

## 3. Discussion

In this in vitro study, we showed that neonatal skin fibroblasts induce less phagocytosis and T cell proliferation than adult skin fibroblasts, through the secretion of immunomodulatory products distinct from HLA-G, and phenotypic differences that do not favor immune cell activation. If confirmed in vivo, these findings could hold great promise in the fields of transplantation and regenerative medicine.

Despite contradictory data related to whether or not allogeneic skin fibroblasts are rejected after transplantation [29,30,31,32,33,34,35,36], these cells hold great promises for tissue-engineering and regenerative medicine applications. In the present study, we showed that part of these controversies may be attributed to the age or source of allogeneic fibroblasts, as phagocytosis and T cell proliferation were less elevated in the presence of neonatal foreskin fibroblasts than adult breast or abdominal skin fibroblasts. However, we do not rule out the possible effect of other variables such as sex, body site, cell senescence, and HLA typing in the observed outcomes of this study. Because we had limited or no access to adult foreskin, neonatal abdominal and breast skin samples, young adult skin, or blood from males and infants, we were unable to address the role of most of these variables. Nonetheless, while our study shows that adult and neonatal skin fibroblasts elicit different T cell proliferative responses, a previous study has reported no significant change [45], suggesting that sex, age, and body site may influence immune responses. HLA typing was also not available for most cell populations we used, and we thus cannot assert that the lack of T cell proliferation we observed was not due to a lack of HLA mismatch between donors. However, because we used 15 different co-culture combinations involving one of six unrelated neonatal skin fibroblast donors and one of four unrelated PBMC donors, it is very unlikely that the consistent reduction we observed in T cell proliferation was due to a lack of HLA mismatch. Due to the extensive degree of HLA polymorphism, the likelihood of finding a well-matched, unrelated donor even when considering HLA-A, HLA-B, and HLA-DR only is very low [46,47]. If our findings are confirmed in vivo, a wide range of transplantable tissue-engineered constructs could be produced from neonatal fibroblasts, including skin, blood vessel, and heart valve substitutes or nerve conduits [1,48,49,50,51]. Importantly, allogeneic neonatal fibroblasts could serve as a universal cell source in transplantation, and be used in combination with biomaterials to accelerate the treatment of patients with critical injuries.

Phagocytosis and antigen presentation are critical in alloantigen recognition [37]. Herein, we showed that PBMC-derived APCs do less phagocytosis when co-cultured with neonatal but not adult skin fibroblasts. Although we did not explore the reasons for these differences, we suspect that fibroblasts derived from neonatal tissues would repress the phagocytic activity or maturation of APCs, through an increased expression of don’t-eat-me signals, such as CD47 [52,53] or CD200 [54]. A previous study has indeed recently shown that specific pathological contexts could induce CD47 expression in skin fibroblasts [55]. However, whether neonatal tissue microenvironment specifically promotes expression of don’t-eat-me signals remains to be addressed. The differences observed in phagocytic activity could also stem from age differences between adult and neonatal skin fibroblasts, since aging cells express increasing levels of senescence markers [56,57], which could serve as triggering signals for phagocytosis.

Alloresponsive T cell activation results in a rapid allograft rejection [37]. In our study, neonatal but not adult skin fibroblasts were shown to reduce T cell proliferation, and this effect was partially mediated through a paracrine mechanism. Thus, future experiments will be necessary to identify the factors that contribute to this effect. It is also likely that the molecules expressed at the cell surface of neonatal skin fibroblasts are involved as well. For example, neonatal skin fibroblasts could express inhibitory ligands, such as PD-L1, that delays allograft rejection through the blockade of T cell activity [58,59]. Accordingly, these aspects could be investigated in the future using proteomic analyses and inhibition experiments.

Previous studies have shown that fibroblasts are heterogeneous across tissues and can switch from steady to activated states in response to external stimuli [21,60]. In line with these studies, our data indicate that neonatal skin fibroblasts can change their phenotype after an inflammatory priming. These cells indeed expressed lower levels of B7-H3 and class II HLA molecules, which are involved in T cell activation [44,61], and increased levels of soluble HLA-G, a molecule usually found in fetal tissues that inhibits the activity of both innate and adaptive immune cells during pregnancy [43]. To confirm whether a reduction in class II HLA molecules could mitigate T cell proliferation, it would be interesting to test the effect of a class II HLA neutralizing antibody in adult skin fibroblast–T cell co-cultures in a subsequent study. Moreover, it is possible that, in response to IFNγ and IL-2 priming, neonatal fibroblasts could reduce the expression of co-stimulatory molecules involved in immune activation, such as CD80 or CD86, as other fibroblasts do in certain pathological contexts [62].

The finding that different subsets of fibroblasts would express more class II HLA molecules than others after IFNγ priming suggests that some fibroblast populations might act as non-professional antigen presenting cells, as previously reported in the heart [63]. In the future, it would be interesting to look at the sensitivity of neonatal and adult skin fibroblasts to IFNγ, through the activation of the IFNγ-dependent major histocompatibility complex class II transactivator type IV (CIITA IV) promoter, the master regulator of class II HLA expression [64].

From an overall clinical perspective, the use of allogeneic neonatal skin fibroblasts is appealing, as they are accessible from leftover tissues after surgery (e.g., foreskin circumcision or polydactyly corrective surgery), and their potential immunogenicity seems to be low according to our data. One direct application of these cells could be in combination with autologous epithelial cells for the production of skin substitutes to treat severe skin injuries. Previous studies from our laboratory have shown that the grafting of skin substitutes containing both autologous keratinocytes and allogeneic fibroblasts does not compromise graft survival in immunocompetent mice [65]. Incorporating allogeneic fibroblasts into such chimeric skin substitutes could be particularly interesting for the treatment of burn patients, as these patients live years after trauma with abnormally high levels of IFNγ and IL-2 [66], two cytokines that were shown to be responsible for reduced class II HLA molecules and B7-H3 expression in neonatal skin fibroblasts in this study. Even though we have not investigated how long these neonatal cells could maintain their interesting properties after grafting, we suspect that they would remain long enough to allow the reestablishment of the cutaneous barrier, before being eventually cleared and replaced by host fibroblasts, as shown in previous studies using Apligraf^®^ or StrataGraft^®^, two different skin substitutes made from neonatal skin fibroblasts, to treat severe wounds in humans [67,68]. Indeed, patients grafted with Apligraf^®^ were shown to heal in 4 weeks on average, a timeframe that corresponded to allogeneic DNA persistence in patients, suggesting that neonatal skin fibroblasts are maintained long enough to promote tissue repair before being eventually cleared. In conclusion, we believe that, due to their potential immunoprivileged status, allogeneic neonatal fibroblasts represent a promising cell source for the production of tissues and grafts destined for treatment of patients with severe injuries.

## 4. Materials and Methods

### 4.1. Skin Fibroblast Isolation and Culture

Skin biopsies were obtained from healthy donors and digested for 16 h at 4 °C in 500 µg/mL thermolysin (Sigma, Oakville, ON, Canada). After removing the epidermis, the dermis was digested for 3 h at 37 °C in 0.125 U/mL collagenase H (Roche, Laval, QC, Canada) to extract the skin fibroblasts. The cells (Table 1) were then centrifuged at 300× *g* for 10 min and counted after staining with trypan blue. Viable fibroblasts were plated at 8000 cells/cm^2^ and cultured in Dulbecco’s Modified Eagle Medium (DMEM; Thermo Fisher Scientific, Ottawa, ON, Canada) with 10% fetal bovine serum (FBS; Avantor Seradigm FB Essence, Radnor, PA, USA), 100 U/mL penicillin (Sigma), and 25 μg/mL gentamicin (Gemini Bio, Sacramento, CA, USA). Before reaching confluency, the cells were harvested after incubation in trypsin (Gibco, Grand Island, NY, USA) and frozen in FBS with 10% DMSO (Sigma).

### 4.2. Peripheral Blood Mononuclear Cell Isolation, Culture, and Differentiation

Blood samples were retrieved from healthy donors in sodium citrate-buffered collection tubes (BD Coulter, Mississauga, ON, Canada) and diluted in half in RPMI 1640 (Thermo Fisher Scientific) containing 2 mM EDTA (Sigma). Peripheral blood mononuclear cells (PBMCs) were isolated from buffy coats after Ficoll-Paque plus (Sigma) gradient centrifugation at 350× *g* for 20 min. Following red blood cell lysis using the RBC lysis buffer (Biolegend, San Diego, CA, USA) and trypan blue staining, PBMCs (Table 1) were counted and frozen in autologous serum with 10% DMSO (Sigma). PBMCs were thawed and cultured in RPMI 1640 (Thermo Fisher Scientific) supplemented with 5% heat-inactivated human AB serum (Sigma), 0.1 mM β-mercaptoethanol (Thermo Fisher Scientific), and a solution containing 100 U/mL penicillin and 100 µg/mL streptomycin (Thermo Fisher Scientific). After 3 h, non-adherent cells, mostly composed of T cells, were collected and cultured separately in PBMC medium supplemented with 200 U/mL IL-2 (StemCell Technologies, Vancouver, BC, Canada). Adherent cells, mostly composed of APCs, were cultured in PBMC medium supplemented with 1000 U/mL Granulocyte-macrophage colony-stimulating factor (GM-CSF) and 250 U/mL IL-4 (both from R&D, Minneapolis, MN, USA). After 4–6 days in culture, immature APCs became non-adherent. The phenotypes of both T cells and immature APCs within PBMC cultures were assessed by flow cytometry. T cells and APCs were not sorted or purified in order to keep a certain immune cell heterogeneity, as it would be in vivo.

### 4.3. Phagocytosis Assay

Subconfluent monolayers of passage 1 or 2 fibroblasts were labeled for 20 min at 37 °C in 6-well plates with 2.5 µM 3,3′-dioctadecyloxacarbocyanine perchlorate (DiO, Thermo Fisher Scientific), a lipophilic fluorescent probe that integrates into cell membranes. After three washes with phosphate-buffered saline (PBS; Thermo Fisher Scientific), 100,000 immature passage 1 APCs were added to each well and the fibroblast–APC co-cultures were maintained in PBMC medium supplemented with 200 U/mL IFNγ (Peprotech, Cranbury, NJ, USA), to promote APC maturation and antigen presentation. After 5 days, DiO uptake in mature APCs was assessed by immunofluorescence and flow cytometry.

### 4.4. T Cell Proliferation Assay

Subconfluent monolayers of passage 1 to 2 fibroblasts were cultured in 6-well plates with 100,000 immature passage 1 APCs per well in PBMC medium supplemented with 200 U/mL IFNγ (Peprotech) and, in some experiments, with a neutralizing antibody targeting HLA-G (Table 2). After 5 days, 1,000,000 passage 1 T cells, stained with carboxyfluorescein succinimidyl ester (CFSE; Thermo Fisher Scientific), a proliferation tracer, were added to each well. The fibroblast–APC–T cell co-cultures were maintained in PBMC medium supplemented with 200 U/mL IFNγ (Peprotech), 200 U/mL IL-2 (StemCell Technologies) and, in some experiments, with a neutralizing antibody targeting HLA-G (Table 2). Seven days later, the expression of CFSE was evaluated in CD4^+^ and CD8^+^ T cell subsets by flow cytometry. The HLA mismatch between the fibroblasts and the immune cells was unknown, but was presumed when consistent hyperproliferative T cell responses against the same allogeneic fibroblast population were observed across repeated experiments.

### 4.5. Flow Cytometry

Adherent cells were gently detached in a cold PBS solution containing 2 mM EDTA (Sigma) using cell scrapers. Adherent and non-adherent cells were fixed for 20 min at room temperature (RT) using a kit containing paraformaldehyde (eBioscience, Waltham, MA, USA). They were incubated for 20 min at 4 °C in a PBS solution containing 0.5 mM EDTA (Sigma), 2% human serum albumin (Sigma), 5 µg/mL polyclonal human immunoglobulins (Sigma) and conjugated primary antibodies (Table 2). After three washes with PBS, the cells were filtered through a 100 µm porosity membrane and analyzed using a FACSMelody cytometer (BD, Mississauga, ON, Canada). Data were plotted on a biexponential scale and analyzed using FlowJo (v10.7.0, LLC, BD).

### 4.6. Immunofluorescence

The cells were fixed in 3.7% formaldehyde (ACP Chemicals, St-Leonard, QC, Canada) for 20 min at RT and rinsed three times with PBS. After 30-minute blocking with a PBS solution containing 2% human serum albumin (Sigma), the cells were incubated for 1 h at RT in the blocking solution containing conjugated primary antibodies (Table 2). Cells were then rinsed three times with PBS and nuclei were stained for 5min at RT in 0.5 µg/mL Hoechst 33,258 (Sigma). After three washes in PBS, the cells were visualized using an LSM700 confocal microscope (Zeiss, Toronto, ON, Canada).

### 4.7. Western Blots

Western blots were conducted under reducing-denaturing conditions with 8 µg protein per well in 10% acrylamide gels. Migration was carried out for 4 h at RT at 80 V. Protein transfer was then conducted for 2 h at 4 °C at 100 V on a nitrocellulose membrane using a buffer containing 5% methanol (Fisher, Mississauga, ON, Canada). The membranes were then stained with Ponceau Red and blocked with a tris-buffered saline containing 0.5% Omnipur polyoxyethylene (20) monolaurate (Millipore, St. Louis, MO, USA) and 5% (*w*/*v*) non-fat powdered milk (Biobasic, Markham, ON, Canada). The membranes were incubated with the primary antibodies overnight at 4 °C, washed 5 times for 10 min and incubated with the secondary antibodies for 1 h at RT (Table 2). The membranes were washed 5 times for 20 min, before the target proteins were revealed with SuperSignal West Pico Plus Chemiluminescent Substrate (Thermo Fisher Scientific) and imaged with the Fusion Fx7 imager (Montreal Biotech, Dorval, QC, Canada). Densitometry quantifications were carried out using ImageJ (v1.53j).

### 4.8. Statistics

Statistical analyses and data representation were executed with R studio (v1.4.1106) from the mean values of technical replicates. All experiments were repeated with at least 3 fibroblast and 2 PBMC populations, which were considered random factors in linear mixed model analyses. Variables such as cell source (adult or neonatal) or stimulation (IFNγ/IL-2 or vehicle) were considered fixed factors in all statistical analysis. Homoscedasticity and normality assumptions were analyzed in all datasets, looking at the distribution of the residuals. When the assumptions were met, parametric tests for paired samples (ANOVA and type II Wald chi-square test) were carried out using the RVAideMemoire package. Post-tests (Tukey and Kenward–Roger) were carried out when significant (*p* < 0.05) single-factor effects were detected in global tests. Plots were generated using the ggplot2 package.

## Figures and Tables

**Table 1:** Cell populations.

Cell Type	Identifier	Source	Sex	Age	Code	Color
Fibroblast	FAabF2146X	Abdomen	F	46	ASF1	khaki
	FAkI2064X	Abdomen	F	64	ASF2	coral
	FArJ2050X	Abdomen	F	50	ASF3	brown
	FAyE2147X	Abdomen	F	47	ASF4	red
	FAyB2143X	Abdomen	F	43	ASF5	pink
	FMaaH1369X	Breast	F	69	ASF6	violet
	FMqC1245X	Breast	F	45	ASF7	yellow
	FPEccG170.1Y	Foreskin	M	<1	NSF1	turquoise
	FPEgI10.1Y	Foreskin	M	<1	NSF2	sky blue
	FPEsE130.1Y	Foreskin	M	<1	NSF3	dark green
	FPEvC200.1Y	Foreskin	M	<1	NSF4	navy blue
	FPEzE130.1Y	Foreskin	M	<1	NSF5	light green
	FPEeeH170.2Y	Foreskin	M	<1	NSF6	grey
PBMC	SAabF2146XT	Blood	F	46	T1	white
	SAabF2146XA	Blood	F	46	APC1	white
	SAkI2064XT	Blood	F	64	T2	black
	SAkI2064XA	Blood	F	64	APC2	black
	SArJ2050XT	Blood	F	50	T3	orange
	SArJ2050XA	Blood	F	50	APC3	orange
	SAyE2147XT	Blood	F	47	T4	lilac
	SAyE2147XA	Blood	F	47	APC4	lilac

Abbreviations: PBMC, peripheral blood mononuclear cell; F, female; M, male; T, T cell; APC, antigen presenting cell; ASF, adult skin fibroblast; NSF, neonatal skin fibroblast.

**Table 2:** Antibodies.

Use	Target	Clone	Specie	Conjugate	Supplier	Cat. #	Dilution
FC	CD3	UCHT-1	Ms	PE/Cy7	BD, Mississauga, CA, USA	563423	1/20
	CD3	HIT3a	Ms	BB700	BD	742207	1/20
	CD4	OKT4	Ms	PE	Biolegend, San Diego, CA, USA	317410	1/20
	CD8	SK1	Ms	PerCP	Biolegend	344708	1/20
	CD11c	3.9	Ms	FITC	Biolegend	301604	1/20
	CD14	M5E2	Ms	PE	Biolegend	301806	1/20
	CD45	2D1	Ms	PE	Biolegend	368510	1/20
	HLADR	L243	Ms	APC	Biolegend	307610	1/20
	HLADR	L243	Ms	PE/Cy7	BD	560651	1/20
N	HLA-G	87G	Ms	-	Thermofisher, Ottawa, ON, Canada	MA110356	2 µg/mL
IF	CD45	2D1	Ms	PE	Biolegend	368510	1/200
WB	HLAABCE	TP2599SF	Ms	-	Novus Bio, Littleton, CO, USA	NBP268006	1/500
	HLADPDQDR	CR3/43	Ms	-	Abcam, Cambridge, MA, USA	ab7856	1/500
	B7-H3	D9M2L	Rb	-	Cell Signaling, Cambridge, MA, USA	14058S	1/1000
	α-Tubulin	DM1A	Ms	-	Sigma, St. Louis, MO, USA	T9026	1/2000
	Rabbit	-	Gt	HRP	Invitrogen, Carlsbad, CA, USA	62-6120	1/5000
	Mouse	-	Gt	HRP	Invitrogen	62-6520	1/5000

Abbreviations: Ms, mouse; Rb, rabbit; Gt, goat; PE, phycoerythrin; BB700, BD Horizon Brilliant Blue 700; PerCP, peridinin chlorophyll; FITC, fluorescein isothiocyanate; APC, allophycocyanin; PE/Cy7, phycoerythrin cyanin 7; HRP, horseradish peroxidase; FC, flow cytometry; N, neutralization; IF, immunofluorescence; WB, Western blotting.

## Data Availability

Data will be made available upon request to the corresponding author.

## Supplementary Materials

The following supporting information can be downloaded at: https://www.mdpi.com/article/10.3390/ijms25136965/s1.

## Abbreviations

APC: antigen-presenting cell; ASF, adult skin fibroblast; CEA, cultured epithelial autograft; CFSE, carboxyfluorescein succinimidyl ester; DiO, 3,3′-dioctadecyloxacarbocyanine perchlorate; FSC, foward scatter; HLA, human leukocyte antigen; IFN, interferon; IgG, immunoglobulin; IL, interleukin; NSF, neonatal skin fibroblast; moDC, monocyte-derived dendritic cell; PBMC, peripheral blood mononuclear cell; PBS, phosphate-buffered saline; SN, supernatant; SSC, side scatter.

## References

[1] Germain L., Larouche D., Nedelec B., Perreault I., Duranceau L., Bortoluzzi P., Beaudoin Cloutier C., Genest H., Caouette-Laberge L., Dumas A. (2018). Autologous Bilayered Self-Assembled Skin Substitutes (SASSs) as Permanent Grafts: A Case Series of 14 Severely Burned Patients Indicating Clinical Effectiveness. Eur. Cells Mater..

[2] Burke J.F., Yannas O.V., Quinby W.C., Bondoc C.C., Jung W.K. (1981). Successful Use of a Physiologically Acceptable Artificial Skin in the Treatment of Extensive Burn Injury. Ann. Surg..

[3] Yannas I.V., Burke J.F., Orgill D.P., Skrabut E.M. (1982). Wound Tissue Can Utilize a Polymeric Template to Synthesize a Functional Extension of Skin. Science.

[4] Yannas I.V., Orgill D.P., Burke J.F. (2011). Template for Skin Regeneration. Plast. Reconstr. Surg..

[5] Downer M., Berry C.E., Parker J.B., Kameni L., Griffin M. (2023). Current Biomaterials for Wound Healing. Bioengineering.

[6] Sadeghianmaryan A., Ahmadian N., Wheatley S., Alizadeh Sardroud H., Nasrollah S.A.S., Naseri E., Ahmadi A. (2024). Advancements in 3D-Printable Polysaccharides, Proteins, and Synthetic Polymers for Wound Dressing and Skin Scaffolding—A Review. Int. J. Biol. Macromol..

[7] Brown J.B., McDowell F. (1942). Massive Repairs of Burns With Thick Split-Skin Grafts. Ann. Surg..

[8] Gibson T., Medawar P.B. (1943). The Fate of Skin Homografts in Man. J. Anat..

[9] Rapaport F.T., Thomas L., Converse J.M., Lawrence H.S. (1960). The Specificity of Skin Homograft Rejection in Man. Ann. N. Y. Acad. Sci..

[10] Burt A.M., Pallett C.D., Sloane J.P., O’Hare M.J., Schafler K., Yardeni P., Eldad A., Clarke J.A., Gusterson B.A. (1989). Survival of Cultured Allografts in Patients with Burns Assessed with Probe Specific for Y Chromosome. Br. Med. J..

[11] Climov M., Matar A.J., Farkash E.A., Medeiros E., Qiao J., Harrington E., Gusha A., Al-Musa A., Sachs D.H., Randolph M. (2016). Survival of Allogeneic Self-Assembled Cultured Skin. Transplantation.

[12] Dvorak H.F., Mihm M.C., Dvorak A.M., Barnes B.A., Manseau E.J., Galli S.J. (1979). Rejection of First-Set Skin Allografts in Man. the Microvasculature Is the Critical Target of the Immune Response. J. Exp. Med..

[13] Larsen C.P., Steinman R.M., Witmer-Pack M., Hankins D.F., Morris P.J., Austyn J.M. (1990). Migration and Maturation of Langerhans Cells in Skin Transplants and Explants. J. Exp. Med..

[14] He C., Schenk S., Zhang Q., Valujskikh A., Bayer J., Fairchild R.L., Heeger P.S. (2004). Effects of T Cell Frequency and Graft Size on Transplant Outcome in Mice. J. Immunol..

[15] Rapaport F.T., Dausset J. (1983). Behavior of HLA-Compatible and Incompatible Skin Allografts in Human Recipients Preimmunized with Pooled Leukocyte Extracts Obtained from Randomly Selected Donors. Transplantation.

[16] Boyce S.T., Kagan R.J., Greenhalgh D.G., Warner P., Yakuboff K.P., Palmieri T., Warden G.D. (2006). Cultured Skin Substitutes Reduce Requirements for Harvesting of Skin Autograft for Closure of Excised, Full-Thickness Burns. J. Trauma-Inj. Infect. Crit. Care.

[17] Larouche D., Cantin-Warren L., Desgagné M., Guignard R., Martel I., Ayoub A., Lavoie A., Gauvin R., Auger F.A., Moulin V.J. (2016). Improved Methods to Produce Tissue-Engineered Skin Substitutes Suitable for the Permanent Closure of Full-Thickness Skin Injuries. Biores. Open Access.

[18] Parker J.B., Valencia C., Akras D., DiIorio S.E., Griffin M.F., Longaker M.T., Wan D.C. (2023). Understanding Fibroblast Heterogeneity in Form and Function. Biomedicines.

[19] Driskell R.R., Watt F.M. (2015). Understanding Fibroblast Heterogeneity in the Skin. Trends Cell Biol..

[20] Cavagnero K.J., Gallo R.L. (2022). Essential Immune Functions of Fibroblasts in Innate Host Defense. Front. Immunol..

[21] Buechler M.B., Pradhan R.N., Krishnamurty A.T., Cox C., Calviello A.K., Wang A.W., Yang Y.A., Tam L., Caothien R., Roose-Girma M. (2021). Cross-Tissue Organization of the Fibroblast Lineage. Nature.

[22] Proost P., Vynckier A.K., Mahieu F., Put W., Grillet B., Struyf S., Wuyts A., Opdenakker G., Van Damme J. (2003). Microbial Toll-like Receptor Ligands Differentially Regulate CXCL10/IP-10 Expression in Fibroblasts and Mononuclear Leukocytes in Synergy with IFN-γ and Provide a Mechanism for Enhanced Synovial Chemokine Levels in Septic Arthritis. Eur. J. Immunol..

[23] Farina G.A., York M.R., Di Marzio M., Collins C.A., Meller S., Homey B., Rifkin I.R., Marshak-Rothstein A., Radstake T.R.D.J., Lafyatis R. (2010). Poly(I:C) Drives Type i IFN- and TGFΒ-Mediated Inflammation and Dermal Fibrosis Simulating Altered Gene Expression in Systemic Sclerosis. J. Investig. Dermatol..

[24] Nickerson P., Steurer W., Steiger J., Zheng X., Steele A.W., Strom T.B. (1994). Cytokines and the Th1/Th2 Paradigm in Transplantation. Curr. Opin. Immunol..

[25] Strom T.B., Roy-Chaudhury P., Manfro R., Xheng X.X., Nickerson P.W., Wood K., Bushell A. (1996). The Th1/Th2 Paradigm and the Allograft Response. Curr. Opin. Immunol..

[26] Xu Z., Chen D., Hu Y., Jiang K., Huang H., Du Y., Wu W., Wang J., Sui J., Wang W. (2022). Anatomically Distinct Fibroblast Subsets Determine Skin Autoimmune Patterns. Nature.

[27] Gao L., Yu Q., Zhang H., Wang Z., Zhang T., Xiang J., Yu S., Zhang S., Wu H., Xu Y. (2021). A Resident Stromal Cell Population Actively Restrains Innate Immune Response in the Propagation Phase of Colitis Pathogenesis in Mice. Sci. Transl. Med..

[28] Vancheri C., Mastruzzo C., Tomaselli V., Sortino M.A., D’Amico L., Bellistrí G., Pistorio M.P., Salinaro E.T., Palermo F., Mistretta A. (2001). Normal Human Lung Fibroblasts Differently Modulate Interleukin-10 and Interleukin-12 Production by Monocytes: Implications for an Altered Immune Response in Pulmonary Chronic Inflammation. Am. J. Respir. Cell Mol. Biol..

[29] Hultman C.S., Brinson G.M., Siltharm S., DeSerres S., Cairns B.A., Peterson H.D., Meyer A.A. (1996). Allogeneic Fibroblasts Used to Grow Cultured Epidermal Autografts Persist in Vivo and Sensitize the Graft Recipient for Accelerated Second-Set Rejection. J. Trauma-Inj. Infect. Crit. Care.

[30] Farrokhi A., Rahavi M., Jo S., Jalili R., Lim C.J., Ghahsary A., Reid G.S. (2022). Inflammatory Immune Responses Trigger Rejection of Allogeneic Fibroblasts Transplanted into Mouse Skin. Cell Transplant..

[31] Hansbrough J.F., Doré C., Hansbrough W.B. (1992). Clinical Trials of a Living Dermal Tissue Replacement Placed beneath Meshed, Split-Thickness Skin Grafts on Excised Burn Wounds. J. Burn Care Rehabil..

[32] Theobald V.A., Lauer J.D., Kaplan F.A., Baker K.B., Rosenberg M. (1993). “Neutral Allografts”—Lack of Allogeneic Stimulation by Cultured Human Cells Expressing MHC Class I and Class II Antigens. Transplantation.

[33] Johnson D.L., Rose M.L., Yacoub M.H. (1997). Immunogenicity of Human Heart Valve Endothelial Cells and Fibroblasts. Transplant. Proc..

[34] Maurer D.H., Collins W.E., Hanke J.H., Van M., Rich R.R., Pollack M.S. (1985). Class II Positive Human Dermal Fibroblasts Restimulate Cloned Allospecific T Cells but Fail to Stimulate Primary Allogeneic Lymphoproliferation. Hum. Immunol..

[35] Samsonov D., Geehan C., Woda C.B., Briscoe D.M. (2012). Differential Activation of Human T Cells to Allogeneic Endothelial Cells, Epithelial Cells and Fibroblasts in Vitro. Transplant. Res..

[36] Shimabukuro Y., Murakami S., Okada H. (1996). Antigen-Presenting-Cell Function of Interferon γ-Treated Human Gingival Fibroblasts. J. Periodontal Res..

[37] Benichou G., Yamada Y., Yun S., Lin C., Fray M., Tocco G. (2011). Immune Recognition and Rejection of Allogeneic Skin Grafts. Immunotherapy.

[38] Lutz M.B., Aßmann C.U., Girolomoni G., Ricciardi-Castagnoli P. (1996). Different Cytokines Regulate Antigen Uptake and Presentation of a Precursor Dendritic Cell Line. Eur. J. Immunol..

[39] Banyer J.L., Halliday D.C.T., Thomson S.A., Hamilton N.H.R. (2003). Combinations of IFN-γ and IL-4 Induce Distinct Profiles of Dendritic Cell-Associated Immunoregulatory Properties. Genes Immun..

[40] Wall M.E., Wani M.C. (1995). Camptothecin and Taxol: Discovery to Clinic—Thirteenth Bruce F. Cain Memorial Award Lecture. Cancer Res..

[41] Savill J. (1997). Recognition and Phagocytosis of Cells Undergoing Apoptosis. Br. Med. Bull..

[42] Ochando J., Ordikhani F., Jordan S., Boros P., Thomson A.W. (2019). Tolerogenic Dendritic Cells in Organ Transplantation. Transpl. Int..

[43] Rouas-Freiss N., LeMaoult J., Moreau P., Dausset J., Carosella E.D. (2003). HLA-G in Transplantation: A Relevant Molecule for Inhibition of Graft Rejection?. Am. J. Transplant..

[44] Chapoval A.I., Ni J., Lau J.S., Wilcox R.A., Flies D.B., Liu D., Dong H., Sica G.L., Zhu G., Tamada K. (2001). B7-H3: A Costimulatory Molecule for T Cell Activation and IFN-γ Production. Nat. Immunol..

[45] Cappellesso-Fleury S., Puissant-Lubrano B., Apoil P.A., Titeux M., Winterton P., Casteilla L., Bourin P., Blancher A. (2010). Human Fibroblasts Share Immunosuppressive Properties with Bone Marrow Mesenchymal Stem Cells. J. Clin. Immunol..

[46] Leffell M.S., Steinberg A.G., Bias W.B., Machan C.H., Zachary A.A. (1994). The Distribution of HLA Antigens and Phenotypes among Donors and Patients in the UNOS Registry. Transplantation.

[47] Ting A., Edwards L.B. (2004). Human Leukocyte Antigen in the Allocation of Kidneys from Cadaveric Donors in the United States. Transplantation.

[48] Boyce S.T., Simpson P.S., Rieman M.T., Warner P.M., Yakuboff K.P., Bailey J.K., Nelson J.K., Fowler L.A., Kagan R.J. (2017). Randomized, Paired-Site Comparison of Autologous Engineered Skin Substitutes and Split-Thickness Skin Graft for Closure of Extensive, Full-Thickness Burns. J. Burn Care Res..

[49] Thibodeau A., Galbraith T., Fauvel C.M., Khuong H.T., Berthod F. (2022). Repair of Peripheral Nerve Injuries Using a Prevascularized Cell-Based Tissue-Engineered Nerve Conduit. Biomaterials.

[50] Galbraith T., Roy V., Bourget J.M., Tsutsumi T., Picard-Deland M., Morin J.F., Gauvin R., Ismail A.A., Auger F.A., Gros-Louis F. (2019). Cell Seeding on UV-C-Treated 3D Polymeric Templates Allows for Cost-Effective Production of Small-Caliber Tissue-Engineered Blood Vessels. Biotechnol. J..

[51] Dubé J., Bourget J.M., Gauvin R., Lafrance H., Roberge C.J., Auger F.A., Germain L. (2014). Progress in Developing a Living Human Tissue-Engineered Tri-Leaflet Heart Valve Assembled from Tissue Produced by the Self-Assembly Approach. Acta Biomater..

[52] Oldenborg P.A., Zheleznyak A., Fang Y.F., Lagenaur C.F., Gresham H.D., Lindberg F.P. (2000). Role of CD47 as a Marker of Self on Red Blood Cells. Science.

[53] Jaiswal S., Chao M.P., Majeti R., Weissman I.L. (2010). Macrophages as Mediators of Tumor Immunosurveillance. Trends Immunol..

[54] Hoek R.H., Ruuls S.R., Murphy C.A., Wright G.J., Goddard R., Zurawski S.M., Blom B., Homola M.E., Streit W.J., Brown M.H. (2000). Down-Regulation of the Macrophage Lineage through Interaction with OX2 (CD200). Science.

[55] Lerbs T., Cui L., King M.E., Chai T., Muscat C., Chung L., Brown R., Rieger K., Shibata T., Wernig G. (2020). CD47 Prevents the Elimination of Diseased Fibroblasts in Scleroderma. JCI Insight.

[56] Tresini M., Pignolo R.J., Allen R.G., Cristofalo V.J. (1999). Effects of Donor Age on the Expression of a Marker of Replicative Senescence (EPC-1) in Human Dermal Fibroblasts. J. Cell. Physiol..

[57] Ressler S., Bartkova J., Niederegger H., Bartek J., Scharffetter-Kochanek K., Jansen-Dürr P., Wlaschek M. (2006). P16INK4A Is a Robust in Vivo Biomarker of Cellular Aging in Human Skin. Aging Cell.

[58] Yang J., Popoola J., Khandwala S., Vadivel N., Vanguri V., Yuan X., Dada S., Guleria I., Tian C., Ansari M.J. (2008). Critical Role of Donor Tissue Expression of Programmed Death Ligand-1 in Regulating Cardiac Allograft Rejection and Vasculopathy. Circulation.

[59] Tanaka K., Albin M.J., Yuan X., Yamaura K., Habicht A., Murayama T., Grimm M., Waaga A.M., Ueno T., Padera R.F. (2007). PDL1 Is Required for Peripheral Transplantation Tolerance and Protection from Chronic Allograft Rejection. J. Immunol..

[60] Plikus M.V., Wang X., Sinha S., Forte E., Thompson S.M., Herzog E.L., Driskell R.R., Rosenthal N., Biernaskie J., Horsley V. (2021). Fibroblasts: Origins, Definitions, and Functions in Health and Disease. Cell.

[61] Nunez G., Ball E.J., Stastny P. (1987). Antigen Presentation by Adherent Cells from Human Peripheral Blood. Correlation between T-Cell Activation and Expression of HLA-DQ and -DR Antigens. Hum. Immunol..

[62] Gunaydin G., Kesikli S.A., Guc D. (2015). Cancer Associated Fibroblasts Have Phenotypic and Functional Characteristics Similar to the Fibrocytes That Represent a Novel MDSC Subset. Oncoimmunology.

[63] Ngwenyama N., Kaur K., Bugg D., Theall B., Aronovitz M., Berland R., Panagiotidou S., Genco C., Perrin M.A., Davis J. (2022). Antigen Presentation by Cardiac Fibroblasts Promotes Cardiac Dysfunction. Nat. Cardiovasc. Res..

[64] Piskurich J.F., Gilbert C.A., Ashley B.D., Zhao M., Chen H., Wu J., Bolick S.C., Wright K.L. (2006). Expression of the MHC Class II Transactivator (CIITA) Type IV Promoter in B Lymphocytes and Regulation by IFN-γ. Mol. Immunol..

[65] Goyer B., Larouche D., Kim D.H., Veillette N., Pruneau V., Bernier V., Auger F.A., Germain L. (2019). Immune Tolerance of Tissue-Engineered Skin Produced with Allogeneic or Xenogeneic Fibroblasts and Syngeneic Keratinocytes Grafted on Mice. Acta Biomater..

[66] Jeschke M.G., Gauglitz G.G., Kulp G.A., Finnerty C.C., Williams F.N., Kraft R., Suman O.E., Mlcak R.P., Herndon D.N. (2011). Long-Term Persistance of the Pathophysiologic Response to Severe Burn Injury. PLoS ONE.

[67] Hu S., Kirsner R.S., Falanga V., Phillips T., Eaglstein W.H. (2006). Evaluation of Apligraf^®^ Persistence and Basement Membrane Restoration in Donor Site Wounds: A Pilot Study. Wound Repair Regen..

[68] Centanni J.M., Straseski J.A., Wicks A., Hank J.A., Rasmussen C.A., Lokuta M.A., Schurr M.J., Foster K.N., Faucher L.D., Caruso D.M. (2011). Stratagraft Skin Substitute Is Well-Tolerated and Is Not Acutely Immunogenic in Patients with Traumatic Wounds: Results from a Prospective, Randomized, Controlled Dose Escalation Trial. Ann. Surg..

