# Supplementary material for: The Human Neonatal Skin Fibroblast, an Available Cell Source for Tissue Production and Transplantation, Exhibits Low Risk of Immunogenicity In Vitro
Source: Int J Mol Sci. 2024 Jun 26;25(13):6965. doi: 10.3390/ijms25136965 (PMC11241615; doi:10.3390/ijms25136965)
Supplement: Supplementary file 1 [file ijms-25-06965-s001.zip › ijms-2990714-supplementary.pdf]

SUPPLEMENTARY DATA

SUPPLEMENTARY MATERIALS AND METHODS

Blood serum extraction

Blood samples were collected using uncoated blood collection tubes (BD) and left for 30 min at room temperature. After coagulation, the samples were centrifuged at 1,500 g for 15 min. The sera were then collected and heat-inactivated at 56 °C for 30 min.

Skin fibroblast isolation and culture

Skin samples were obtained from healthy donors and digested for 16 hours at 4 °C in 500 µg/ml Thermolysin (Sigma). After removing the epidermis with tweezers, the dermis was digested for 3 hours at 37 °C in 0.125 U/ml collagenase H (Roche) to extract the skin fibroblasts. The cells were then centrifuged at 300 g for 10 min and counted after staining with trypan blue. Viable fibroblasts were plated at 8,000 cells/cm^2 and cultured in Dulbecco's Modified Eagle Medium (DMEM) with 10 % fetal bovine serum (FBS) and antibiotics. Before reaching confluency, the cells were harvested after incubation in trypsin (Gibco) and frozen in FBS with 10 % DMSO (Sigma). All fibroblast populations were used at passage 1 or 2 in this study. Their name and source are listed in Supplementary table 1.

Self-assembled dermal matrix production and decellularization

Self-assembled dermal matrices (SDMs) were produced and decellularized according to a previously published protocol (Magne, Demers et al. 2023). Briefly, skin fibroblasts were seeded at 4,000 c/cm^2 and cultured in fibroblast medium supplemented with 50 µg/mL ascorbic acid (Sigma) for 21 days to generate cohesive dermal sheets. SDMs were produced after stacking three dermal sheets together and applying 150 Pa static pressure on top of them for 48 hours to promote dermal sheet fusion. The SDMs were used after five additional days in culture without static pressure in the fibroblast medium supplemented with 50 µg/mL ascorbic acid (Sigma). Decellularized SDMs (DSDMs) were obtained after two cycles of decellularization, each consisting of three

main incubation steps with water for 15-18 hours at RT, PBS supplemented with 50 mM magnesium chloride (Sigma) and 2 U/ml DNAse I (Sigma) for 5 hours at 37°C, and water for 18-21 hours at RT.

Tissue solubilization

SDMs and DSDMs were finely cut, snap-frozen in liquid nitrogen and ground for 30 min using 9-mm stainless beads in a Cryomill MM400 (Retsch). The samples were then incubated for 24 hours at 4 °C in a solution containing 50 mM Tris-HCl (Biobasic) and a commercially-available cocktail of protease and phosphatase inhibitors (Cell Signaling). The tissue extracts were then dialyzed overnight at 4 °C against water using a dialysis membrane with a 10K molecular weight cut-off (Thermofisher), and assayed for protein content using a Pierce BCA kit (Thermofisher).

Flow cytometry

Adherent cells were gently detached in a cold PBS solution containing 2 mM EDTA (Sigma) using cell scrapers. Adherent and non-adherent cells were fixed for 20 min at room temperature (RT) using a commercially available kit (eBioscience). They were incubated for 20 min at 4 °C in a PBS solution containing 0.5 mM EDTA (Sigma), 2 % human serum albumin (Sigma), 5 µg/ml polyclonal human immunoglobulins (Sigma) and conjugated primary antibodies (see Supplementary table 2 for antibody references and dilutions). After three washes with PBS, the cells were filtered through a 100 µm porosity membrane and analyzed using a FACSMelody cytometer (BD). Data were plotted on a biexponential scale and analyzed using FlowJo (v10.7.0, LLC).

Immunofluorescence

The cells were fixed in 3.7 % formaldehyde (ACP Chemicals) for 20 min at RT and rinsed three times with PBS. After a 30-min blocking with a PBS solution containing 2 % human serum albumin (Sigma), the cells were incubated for 1 hour at RT in the blocking solution containing conjugated primary antibodies (see Supplementary table 2 for antibody references and dilutions). The cells were then rinsed three times with

PBS and stained for 5min at RT in 0.5 µg/ml Hoechst 33258 (Sigma). After three washes in PBS, the cells were visualized using an LSM700 confocal microscope (Zeiss).

Protein extraction

Cell cultures were lysed on ice in a PBS solution containing 10 mM sodium deoxycholate (Fisher), 3 mM sodium dodecylsulfate (Biorad), 1 % NP40 (Biobasic), 1 % triton-X-100 (Biorad) and a commercially-available cocktail of protease and phosphatase inhibitors (Cell Signaling). The lysates were sonicated for 15 seconds at 20 % amplitude and centrifuged at 13,000 g for 10 min. Supernatants were then collected and assayed for protein content using a Pierce BCA kit (Thermo Fisher).

Western blots

Western blots were conducted under reducing-denaturing conditions with 8 µg protein per well in 10 % acrylamide gels. Migration was carried out for 4 hours at RT at 80 V. Protein transfer was then conducted for 2 hours at 4 °C at 100 V on a nitrocellulose membrane using a buffer containing 5 % methanol (Fisher). The blots were then stained with Ponceau Red and blocked with a tris-buffered saline containing 0.5 % Omnipur polyoxyethylene (20) monolaurate (Millipore) and 5 % (w/v) non-fat powdered milk (Biobasic). The blots were incubated with the primary antibodies overnight at 4 °C and after several washes with the secondary antibodies for 1 hour at RT (see Supplementary table 2 for antibody references and dilutions). After several washes, target proteins were revealed with SuperSignal West Pico Plus Chemiluminescent Substrate (Thermo Fisher) and imaged with the Fusion Fx7 imager (Montreal Biotech). Densitometry quantifications were carried out using ImageJ (v1.53j).

Immunoglobulin isotype-specific enzyme-linked immunosorbent assay (ELISA)

Ninety-six-well plates were coated overnight at 4°C with 10 µg/ml solubilized tissue extracts diluted in a 0.2 M sodium bicarbonate (Biobasic) solution at pH 9.4. The plates were washed four times in a solution containing 150 mM sodium chloride (Fisher), 25

mM tris (Biobasic) and 0.3 % Brij-35 (Sigma) and blocked for 1 hour at RT in the same washing solution supplemented with 2% bovine serum albumin (Proliant). After four washes, serial two-fold dilutions of the immunized mouse sera were added to each well and incubated for 2 hours at RT. The plates were then rinsed four times with the washing solution, and incubated for 1 hour at RT with horseradish peroxidase conjugated goat anti-mouse isotype specific antibodies (see Supplementary table 2 for antibody references and dilutions). Appropriate working dilutions and isotype specificities of these antibodies were tested in preliminary experiments using purified immunoglobulin isotypes from murine melanoma (Sigma), that were also used as standards in all other assays. The plates were then washed four times and incubated for 20 min at RT in the dark with tetramethylbenzidine (Thermofisher). After adding the sulfuric acid-based stop solution (Thermofisher) in each well, the absorbance was read at 450 nm using a Varioskan Flash microplate reader (Thermofisher).

SUPPLEMLENTARY FIGURES

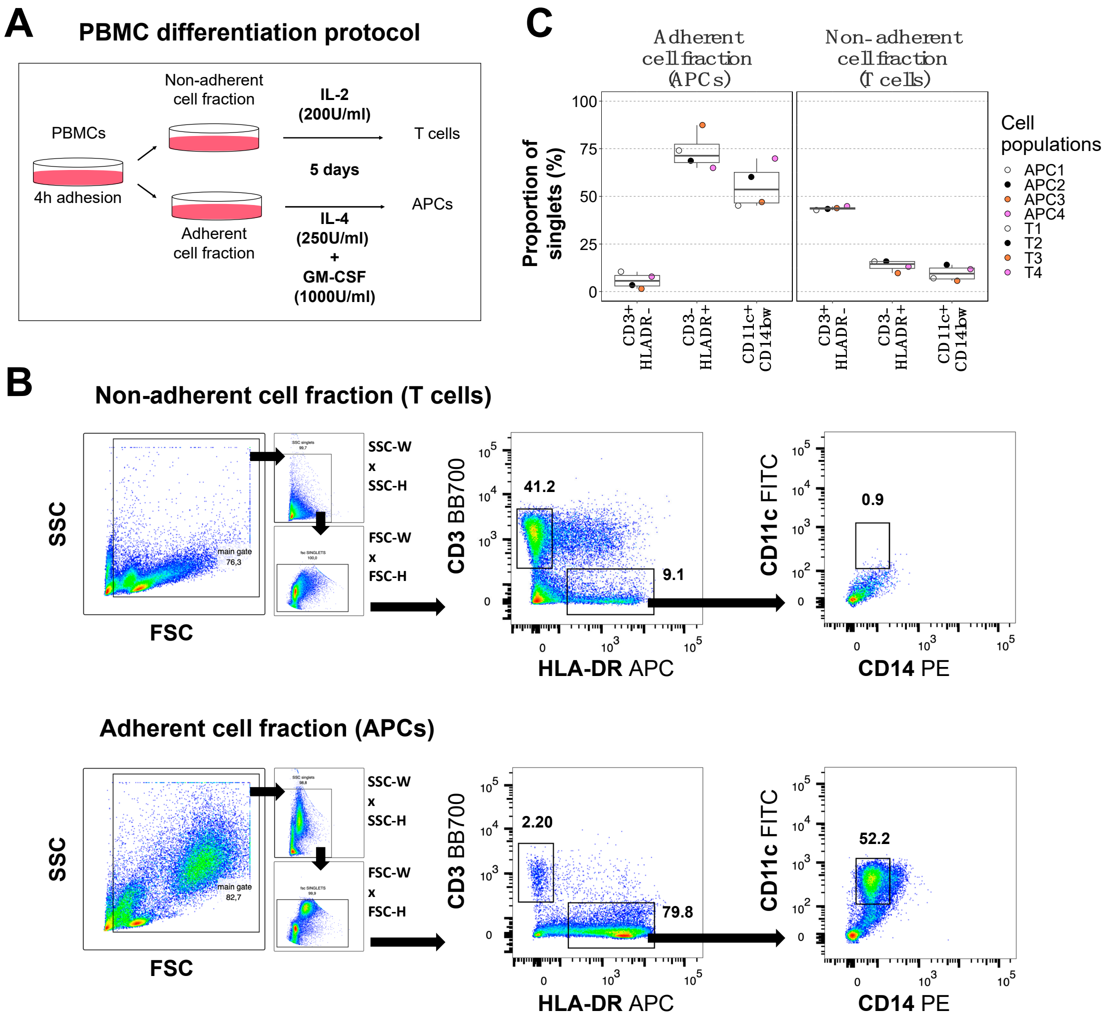

Figure S1. Characterization of peripheral blood mononuclear cell-derived T cells and antigen presenting cells. (A) Schematic of the peripheral blood mononuclear cell (PBMC) differentiation protocol leading to T cell and antigen presenting cell (APC) isolation. **(B)** Cytometric phenotype analysis of the isolated T cells and APCs from non-adherent and adherent cell fractions. Plots, generated on a biexponential scale, show the gating strategy used to exclude doublets and isolate CD3^{+} T cells and HLA-DR^{+} CD11c^{+} CD14^{low} monocyte-derived dendritic cells (moDCs) from the T4 and APC4 populations, respectively. **(C)** Proportion of CD3^{+} HLADR^{-} T cells, CD3^{-} HLADR^{+} APCs and CD3^{-} HLADR^{+} CD11c^{+} CD14^{low} moDCs after PBMC differentiation in both adherent and non-adherent cell fractions. This analysis was performed on N=4 different PBMC populations, and repeated 1 to 2 times for each population. Each point represents the mean value across repeated analyses for each population.

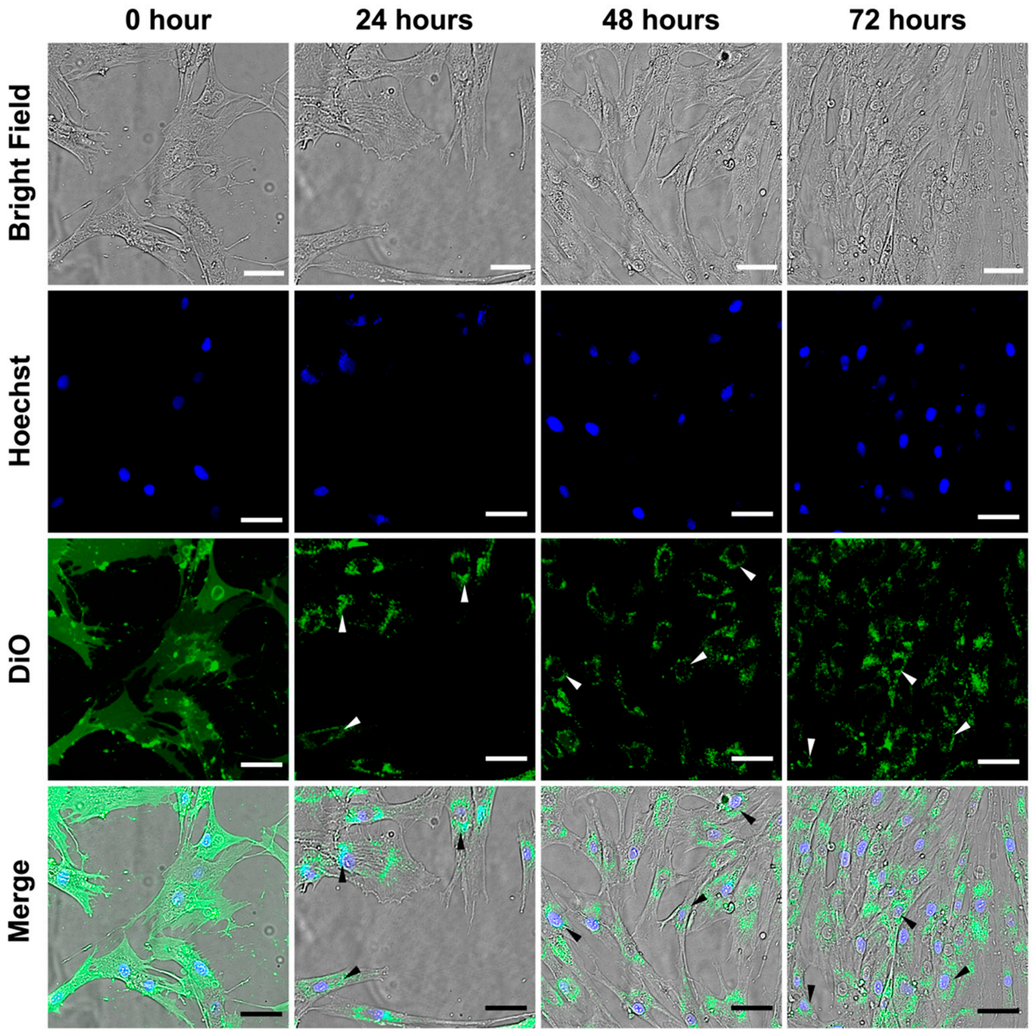

Figure S2. DiO staining is internalized over time in cultured fibroblasts. Fibroblasts were stained with DiO and cultured over 72 hours. Cells were fixed at 0, 24, 48 and 72 hours after DiO staining and labeled with Hoechst. Immunofluorescence and bright field pictures are presented for all time points. Scale bar: 50μm.

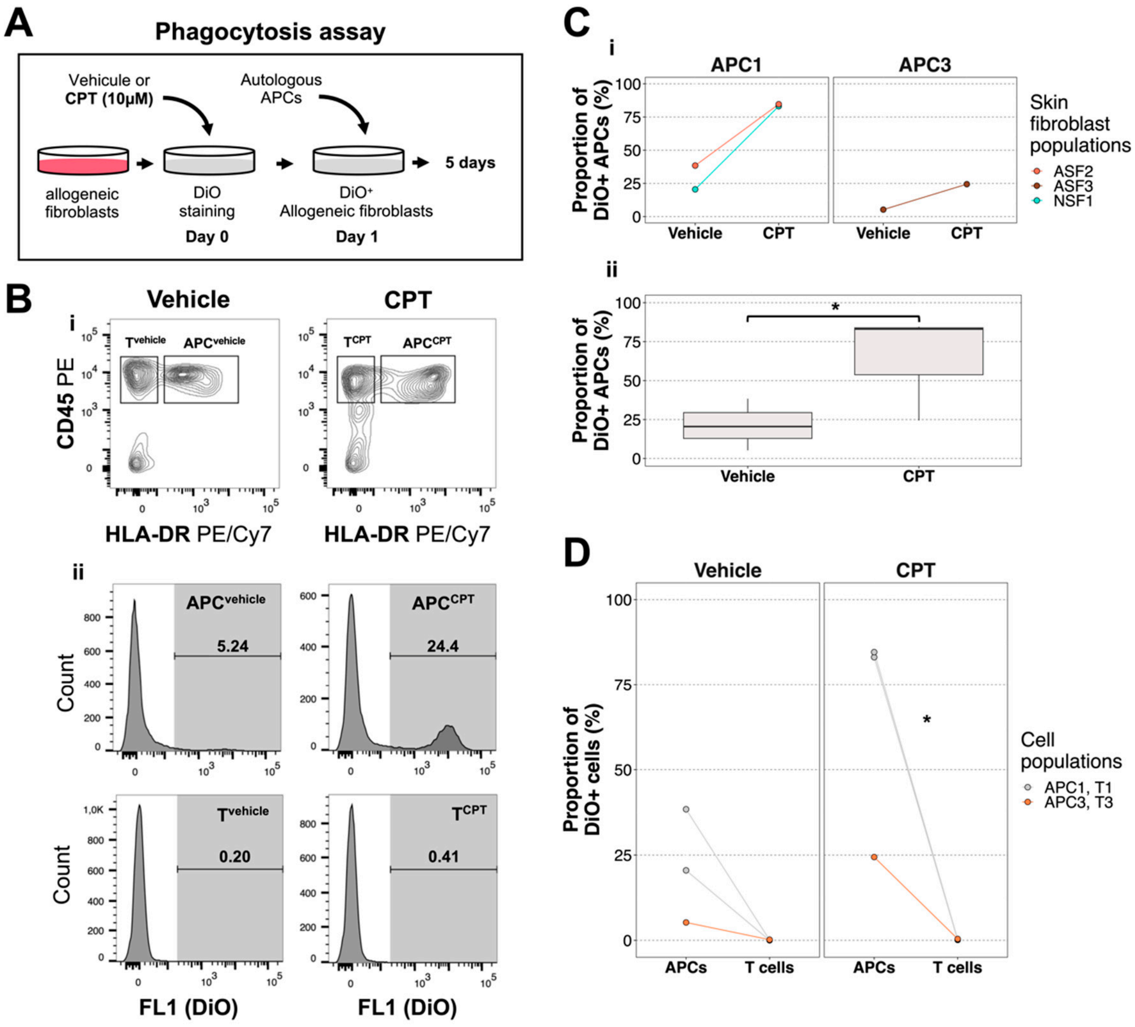

Figure S3. DiO incorporation occurs in antigen presenting cells (APCs), but not in T cells, and is enhanced when APCs are co-cultured with apoptotic cells. (A) Schematic of the experimental design. Camptothecin (CPT), an apoptotic inducer, or vehicle were used to treat DiO$^+$ fibroblasts before cultivation with APCs for 5 days. **(B)** Cytometric analysis of DiO incorporation by APCs and T cells in co-cultures with DiO$^+$ fibroblasts treated with CPT or vehicle. (i) Gating strategy used to isolate APCs and T cells. Plots are generated on a biexponential scale. (ii) Histograms showing DiO incorporation in APCs and T cells after vehicle or CPT treatment. DiO expression is detected across the fluorescent detector channel 1 (FL1). Representative graphs are provided for the ASF3–APC3–T3 co-culture. **(C)** Quantification of the proportion of DiO$^+$ APCs in N=3 different fibroblast–APC co-culture systems. Data are presented in two separate graphs to show (i) values for individual co-culture systems and (ii) grouped values for each condition (vehicle vs CPT). Statistics: paired-T test; * $p < 0.05$. **(D)** Quantification of the proportion of DiO$^+$ APCs and T cells in N=3 fibroblast–moDC co-culture systems (same as in C). Statistics: Wald test and with Kenward-roger's multiple-comparison tests; * $p < 0.05$.

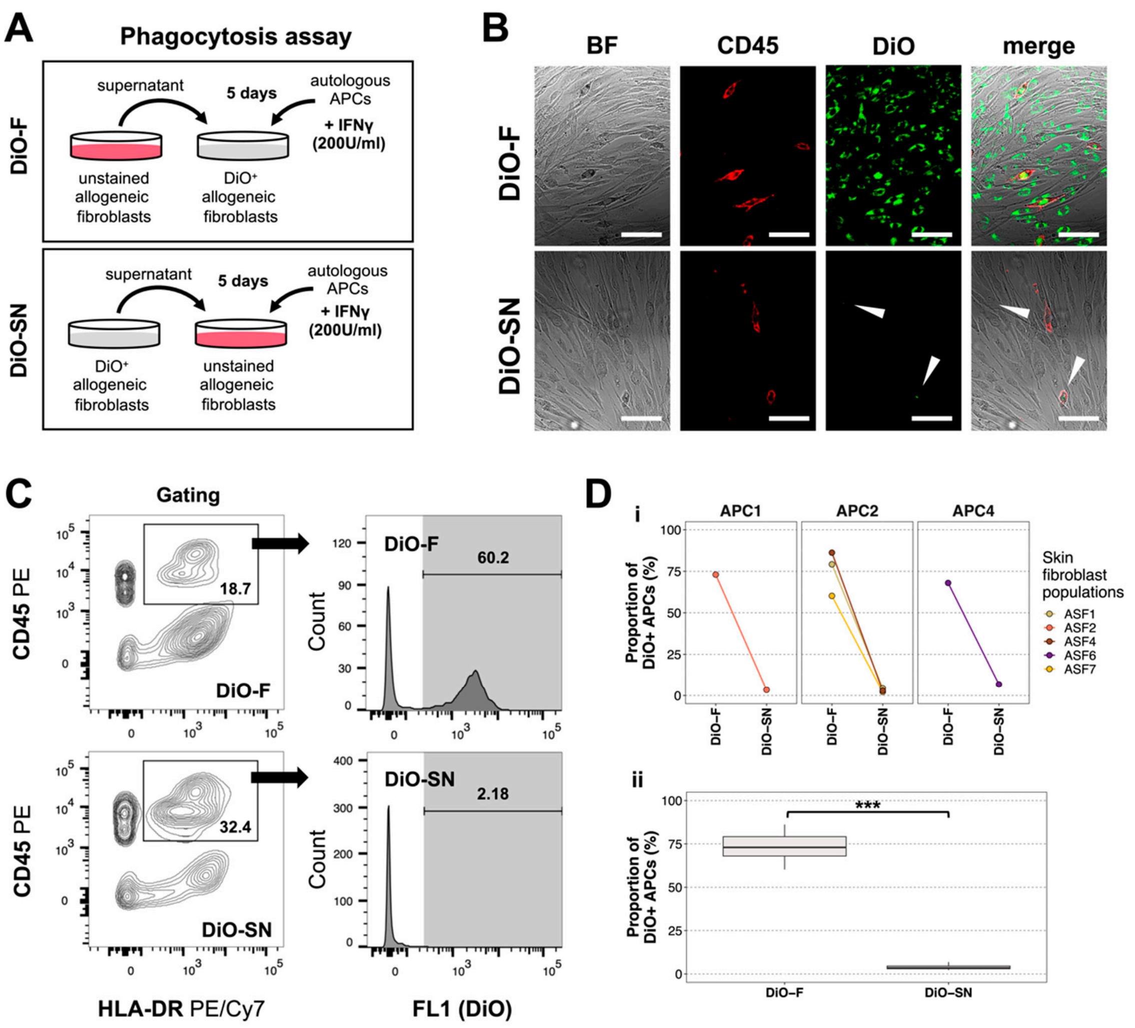

Figure S4. Antigen presenting cells (APCs) cultured with supernatant derived from DiO⁺ fibroblasts are not positive for DiO. (A) Schematic of the experimental conditions tested. DiO-F: APCs were cultured with DiO⁺ fibroblasts and the co-culture was supplemented with supernatant from the same DiO⁻ fibroblast population. DiO-SN: APCs were cultured with DiO⁻ fibroblasts and the co-culture was exposed to supernatant from the same DiO⁺ fibroblast population. **(B)** Immunofluorescence analysis of DiO incorporation in both DiO-F and DiO-SN conditions. Representative pictures are shown for the APC2–ASF7 co-culture. Arrowheads show punctate expression of DiO in the DiO-SN condition. BF: Bright Field. Scale bar: 100μm. **(C)** Cytometric analysis of DiO incorporation for APC2–ASF7 co-culture. DiO expression is detected across the fluorescent detector channel 1 (FL1). Representative graphs of the gating strategy are presented here. Plots are generated on a biexponential scale. **(D)** Quantification of the proportion of DiO⁺ APCs in N=5 different APC–adult skin fibroblast (ASF) co-cultures. Data show (i) values for individual co-cultures and (ii) grouped values for each condition (DiO-F vs DiO-SN). Statistics: paired-T test; *** $p< 0.001$.

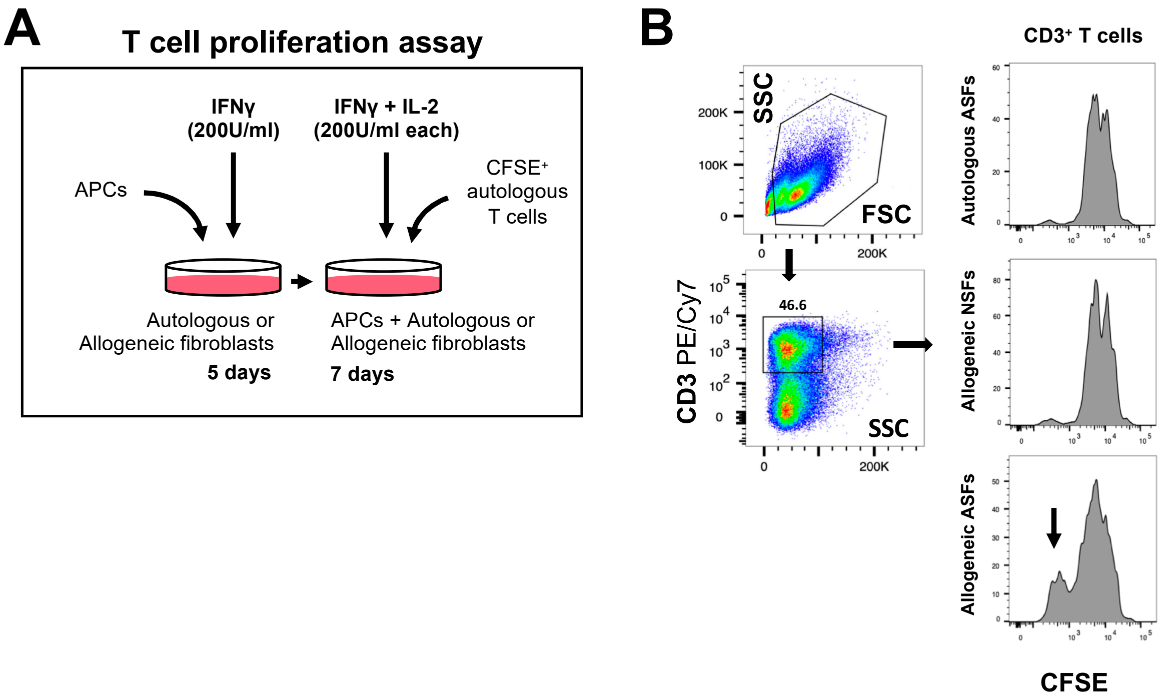

Figure S5. T cell proliferation in autologous and allogeneic co-culture systems. (A) Schematic of the T cell proliferation assay using carboxyfluorescein succinimidyl ester (CFSE) labeling. **(B)** Cytometric analysis of CD3+ T cell proliferation. Left panel: Gating strategy used to isolate CD3+ T cells. Plots are generated on a biexponential scale. Right panel: Histograms showing CFSE expression in CD3+ T cell subsets across three different conditions: T cell and autologous adult skin fibroblasts (ASFs), T cell and allogeneic neonatal skin fibroblast (NSFs), and T cell and allogeneic ASFs. Black arrow shows increased loss of CFSE staining, and thus increased proliferation of T cells, when allogeneic ASFs are used in the co-cultures.

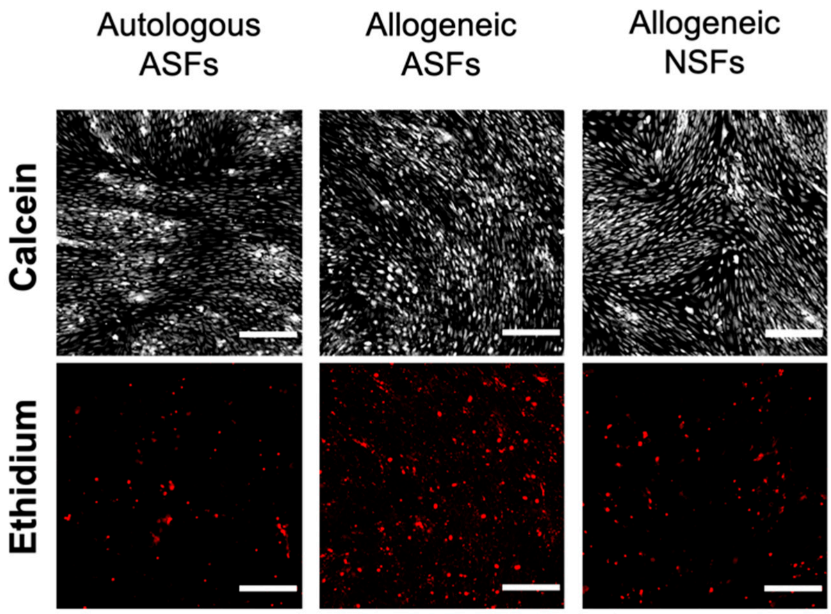

Figure S6. Cell death is higher in ASFs than NSFs, after co-culture with donor-mismatched T cells. ASFs and NSFs were co-cultured with donor-mismatched T cells and stained for Calcein (living cells) and Ethidium (dead cells) using a labelling kit (Invitrogen, L3224). Results are from a single experiment. Scale bar: 300 µm.

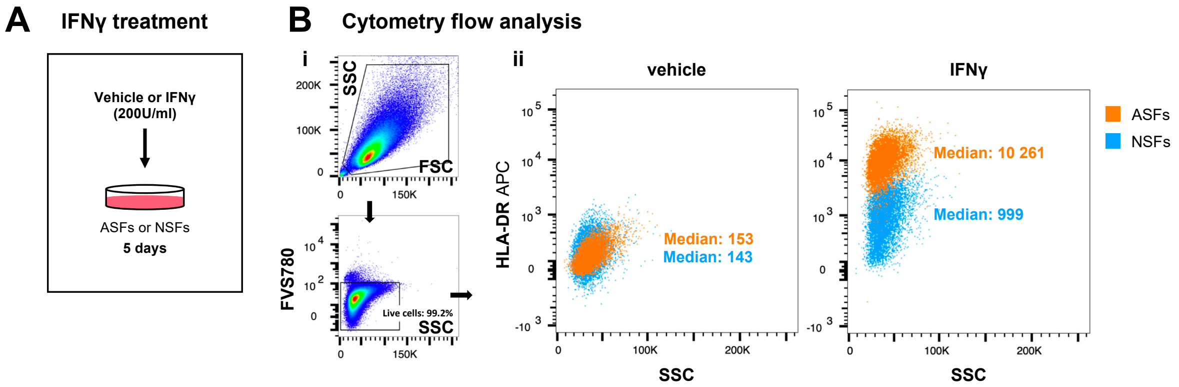

Figure S7. HLA-DR surface expression after interferon gamma (IFNγ) treatment is lower in neonatal (NSFs) than adult (ASFs) skin fibroblasts. (A) Schematic of the experimental design. **(B)** Flow cytometric analysis of HLA-DR surface expression by ASFs and NSFs after vehicle or IFNγ treatment. (i) Gating strategy used to isolate living cells by FVS780 positive cell exclusion. (ii) HLA-DR-SSC plots of ASFs and NSFs after vehicle or IFNγ treatment. Median value intensities for HLA-DR are indicated for each test group.
